# The effect of extra-osseous talotarsal stabilization (EOTTS) to reduce medial knee compartment forces – An in vivo study

**DOI:** 10.1371/journal.pone.0224694

**Published:** 2019-12-12

**Authors:** Lukas Kolodziej, Rodney K. Summers, Michael E. Graham

**Affiliations:** 1 Clinic of Orthopaedics Traumatology and Orthopaedic Oncology, Pomeranian Medical University, Szczecin, Poland; 2 Engineering Center for Orthopaedic Research Excellence (ECORE), Departments of Bioengineering and Orthopaedic Surgery, Colleges of Engineering and Medicine, The University of Toledo, Toledo, OH, United States of America; 3 Department of Foot and Ankle Surgery, Graham International Implant Institute, Macomb, MI, United States of America; Texas State University, UNITED STATES

## Abstract

**Background:**

Excessive hindfoot pronation, talotarsal joint (TTJ) instability, has been attributed to an increase in medial knee compartment pathology. Advocacy for hindfoot realignment has been the subject of research. An internal solution for TTJ instability, extra-osseous talotarsal stabilization (EOTTS), exists but its effect on knee forces is unknown. This is the first study to measure the in vivo forces acting within the medial knee compartment before and after EOTTS. We hypothesized that following EOTTS there should be a reduction of force acting on the medial knee compartment.

**Methods:**

10 fresh frozen cadaver lower extremities exhibiting clinical and radiographic evidence of TTJ instability were evaluated. The proximal femur segment was mounted to a mechanical testing unit. Pressure sensors were placed within the medial knee compartment. A force of 1000 newtons was then applied, and the femur was internally rotated 10 degrees. Measurements were recorded before and after the insertion of a type II EOTTS stent.

**Results:**

Pre-EOTTS resulted in an average of 842 ± 247N acting within the medial knee joint compartment. These forces then decreased to an average of 565 ± 260N (<0.05) following EOTTS, representing an average reduction of force by 32.8%.

**Conclusion:**

EOTTS has been shown to decrease the in vivo forces action within the medial knee compartment. This helps to further illustrate the importance of realigning and stabilizing the hindfoot for the prevention and treatment of chronic knee pain.

## Introduction

Chronic knee pain has a negative impact to both an individual’s lifestyle and overall health. It can lead to a decrease in activity level and be associated with many metabolic diseases such as obesity, diabetes, and heart disease [1]. Furthermore, the burden placed on the elderly population both personally and economically, is of great concern [2]. Year after year, the number of patients developing knee pain continues to increase while the related treatment costs continue to increase [3]. The lifetime risk of developing OA is 44.7% [4].

Most people take thousands of steps every day, resulting in millions of steps per decade. If the forces acting on the medial and lateral compartments of the knee joint are not balanced, this could result in accelerated cartilage degradation within one of the compartments. Scientific evidence suggests that altered lower extremity biomechanics lead to excessive loading of the medial knee compartment during weightbearing activities [5–7]. Many studies have focused on improving lower extremity alignment to reduce the mechanical load on the medial knee compartment[8,9].

Extra-osseous talotarsal stabilization (EOTTS), an improvement over intra-osseous subtalar arthroereisis, is a minimally invasive, conservative surgery that involves the insertion of an extra-osseous titanium stent into the sinus tarsi, a naturally occurring space between the talus and calcaneus. This soft tissue procedure maintains the alignment and stability of the talus on the calcaneus and navicular bones, the talotarsal joint (TTJ), while still allowing a normal range of motion [10]. One of the many positive benefits of EOTTS is the ability to prevent the partial dislocation of the talus on calcaneus. TTJ subluxation leads to instability within the subtalar and talonavicular joints, ultimately creating an overpronation of the hindfoot. The connection between hindfoot misalignment, over-pronation, and the ill-effects to proximal joint structures has been established [11–13].

An important factor in the treatment of knee pain should be the realignment and stabilization of the excessive hindfoot motion. It would make sense that an internal hindfoot stabilization procedure would have a positive effect on the knee. No prior research has evaluated the role or importance of EOTTS as an adjunct option for the prevention or treatment of chronic knee pain. Studies have been performed to evaluate the role of arch supports on knee pain The purpose of this study was to evaluate the difference of *in vivo* forces acting on the medial compartment of the tibiofemoral joint in limbs diagnosed with subtalar joint instability, prior to and post-EOTTS. We theorized that EOTTS would decrease the forces acting on the medial knee compartment through the realignment of the subtalar joint and the joints superior to it. Reducing the load carried by the medial tibiofemoral compartment.

## Materials and methods

In this study we used ten human cadaveric lower limb specimens. Testing on cadaveric knees is necessary because this data could not be obtained in living patients. The University of Toledo Ohio, Engineering Center for Orthopaedic Research Excellence (ECORE) has a long history of performing cadaveric experiments and have established working principles to ensure all ethical issues and legal aspects are covered. ECORE has an Anatomical department which has the authority under Ohio law to use human tissue for educational and research purposes. The director of ECORE is responsible for the ethical issues with regard to the use of human cadaver material. The cadaveric experiment was conducted according to the protocol issued by ECORE and were approved by both our local ethics advisor, Dr. Vijay Goel. None of the transplant donors were from a vulnerable population and all donors or next of kin provided written informed consent that was freely given.

**Fig 1:**
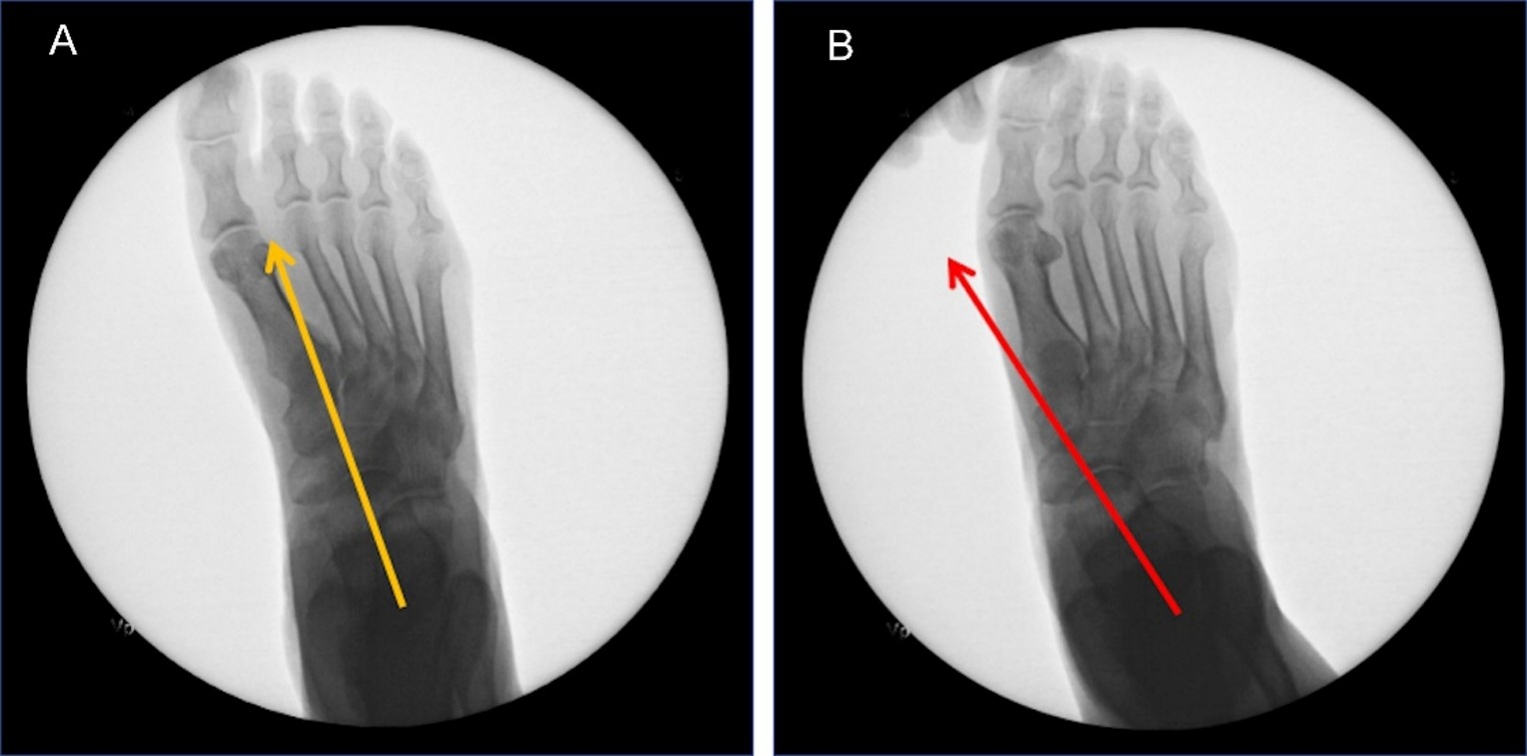
Simulated weightbearing dorsoplantar fluoroscopic imaging.

Ten fresh-frozen human adult cadaveric lower limbs age (49–95 years, mean 66.8 years), two left and eight right, were chosen. The limbs were ethically sourced from two different tissue banks within the United States. Limbs with a previous history of knee or hindfoot surgery, fractures, or pathological conditions of the ankle-hindfoot complex, were excluded. The specimens were allowed to adequately thaw to room temperature before testing. Limbs with normal TTJ pronation, rigid non-reducible hindfoot alignment, or previous history of hindfoot surgery were excluded. Limbs that exhibited a clinical range of motion (ROM) examination, specially TTJ pronation, greater than 6 degrees TTJ pronation were then evaluated via lateral and dorsoplantar (DP) fluoroscopic simulated weightbearing imaging (Fig 1).

The limbs were then dissected free of all soft tissue approximately 10 cm proximal to the knee joint line. The femur was transected to allow for potting in polyester resin for mounting on the MTS Bionix® (MTS, Eden Prairie, MN) apparatus. Care was taken to ensure proper potting where the knee and foot would be in neutral anatomic alignment when inserted into the testing fixture. TekScan® sensors, Model 4000, (TekScan®, Boston, MA) and the I-Scan software (TekScan®) were used for recording pressure measurements within the joints and were calibrated using a four-point calibration curve (10%, 25%, 60%, and 90% of the peak expected load) with a peak expected load of 2000 N, at standard room temperature. A new sensor was used for each specimen.

**Fig 2:**
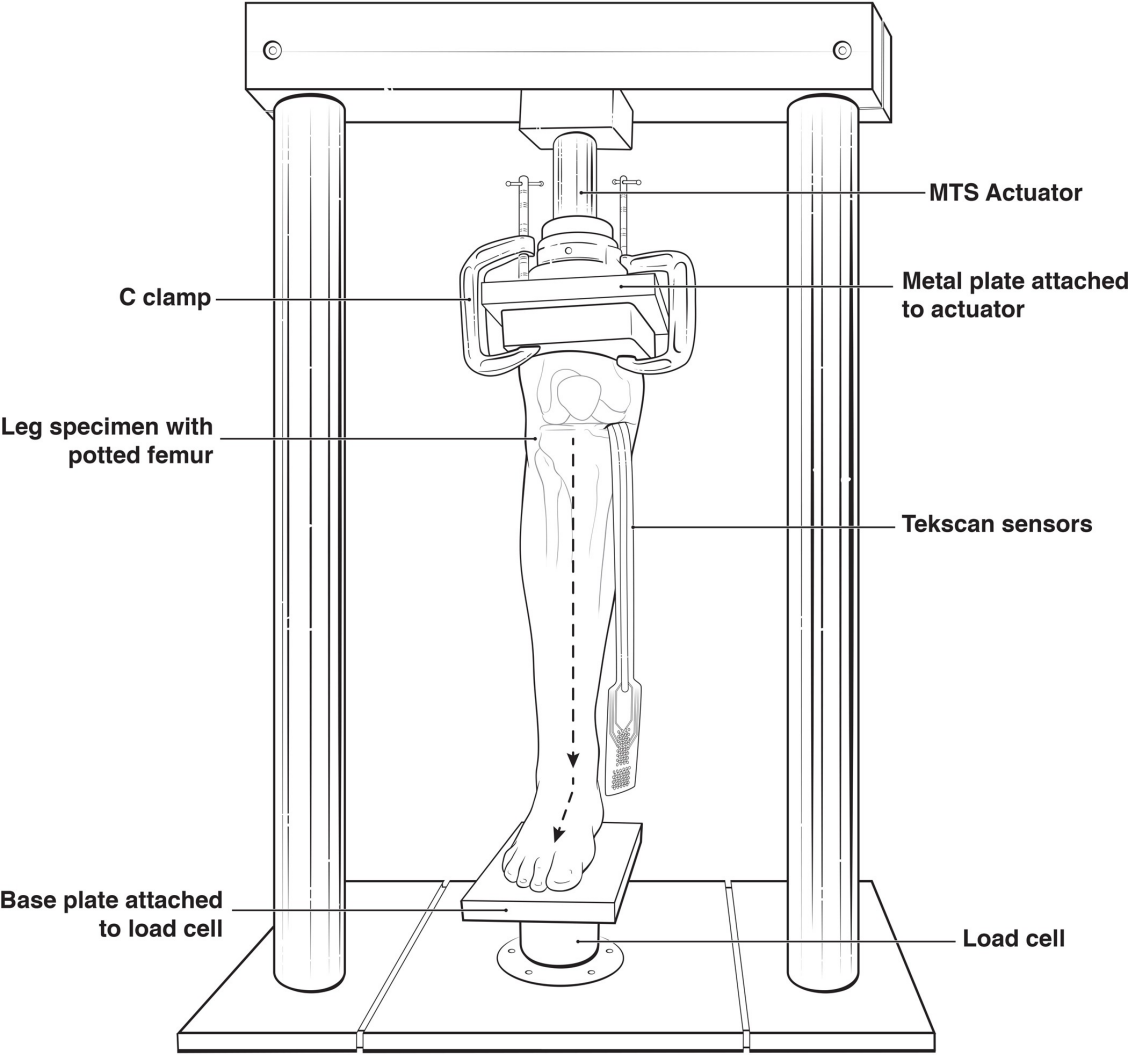
Shows the potted above knee limb with pressure sensor inserted into the medial knee compartment.

The legs, before insertion of the HyProCure II® stent (GraMedica®, Macomb, MI), were mounted to the MTS Bionix® servohydraulic test system machine (Fig 2). Specimens were positioned centrally with the knee and foot in a neutral position. The foot was left free on the load cell. The sensors were inserted into the medial tibiofemoral joint. A minimal incision was made over the medial tibiofemoral joint in an oblique manner. The medial collateral ligament, superficial ligaments, and capsular tissue were carefully dissected to expose the joint only enough for the sensor to be inserted between the femoral condyle and medial tibial compartment.

A 1000 N compressive force at 500 N/min and 10˚ internal rotational at 5˚/min was applied. These forces were used at those rates to slowly but steadily increase the simulated weightbearing force without affecting the placement of the sensor. Axial displacement and angular torque were recorded throughout the load application process. Pressure measurements were collected from the TekScan sensors from the medial knee compartment at the end of each load cycle to compute contact force. The raw pressure data from each measured trial were converted to forces on the joint using I-Scan software.

After testing the “intact” specimens, the MTS machine forces were unloaded. EOTTS was performed using the HyProCure II® stent surgically inserted into the sinus tarsi of each specimen, by a trained and qualified foot surgeon. A 1 ½ to 2 cm incision was created anteroproximal to posterodistal centered over the sinus tarsi using a #15 blade. Superficial blunt dissection over the sinus tarsi was performed utilizing curved Steven’s tenotomy scissors. The interosseous talocalcaneal ligament was decompressed to allow reposition of the talus on the calcaneus and to allow proper insertion of the sinus tarsi stent. A guide wire was placed into the sinus tarsi to ensure proper trial sizing of the stent. A size six trial sizer was inserted deep into both the canalis and sinus portions of the sinus tarsi. A firm dorsolateral pronatory force was applied to the plantar aspects of the 4th and 5th metatarsal necks, to test the amount of TTJ pronation. Subsequent trial sizers were inserted and removed until the desired size was determined for each individual foot. Three size 8 and seven size 9 stents were used for the specimens.

**Fig 3:**
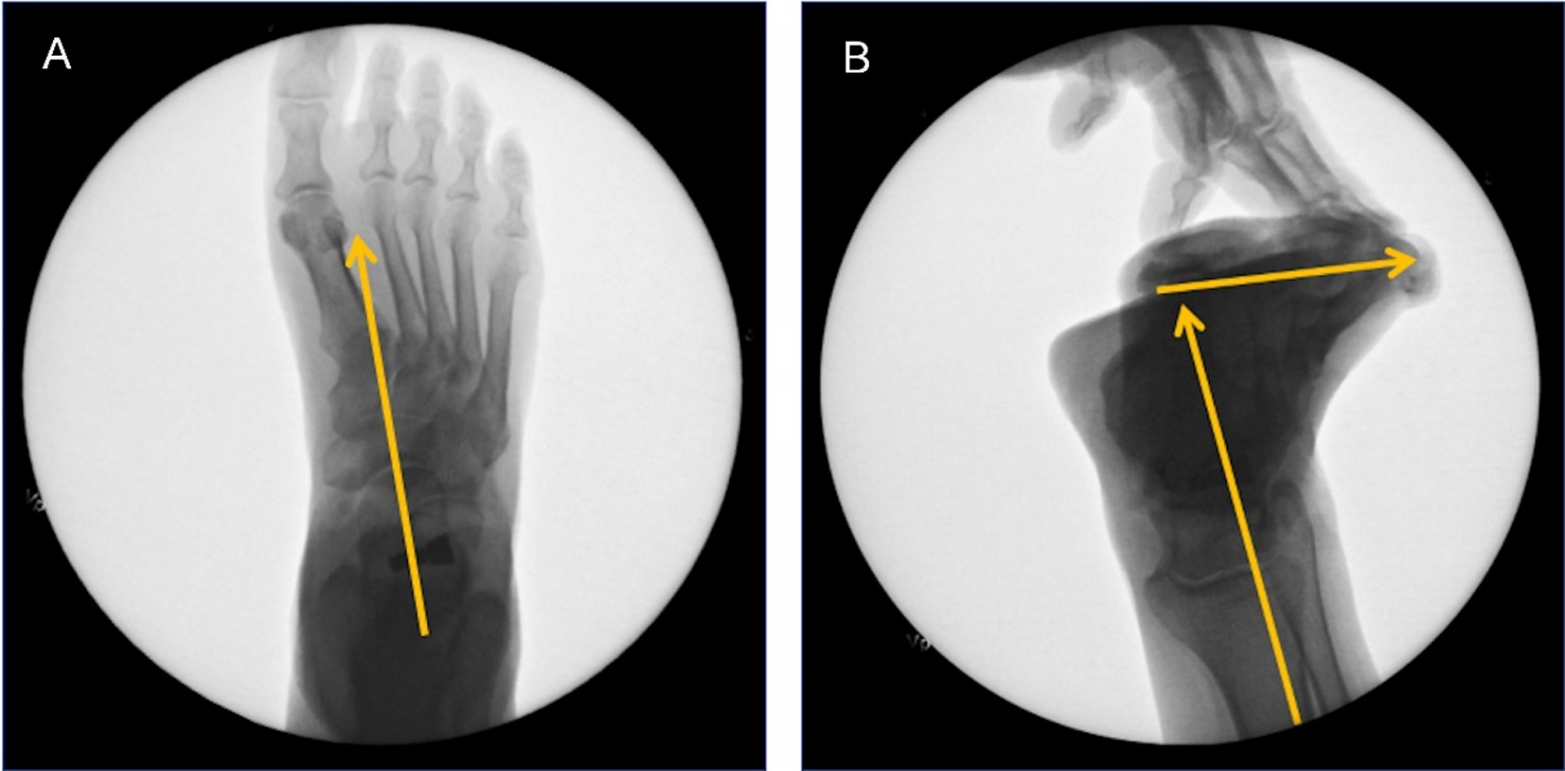
A, B, Simulated weightbearing fluoroscopic images post-EOTTS.

Forced lateral and DP fluoroscopic imaging were taken once the stent was placed to ensure proper placement, and to also verify angular normalization of the talar declination on the lateral view and talar second metatarsal on the DP view (Fig 3A). Furthermore, a forced pronatory pressure was applied to the forefoot to ensure a normal amount of talotarsal joint pronation was still present, i.e. to ensure the correct size stent was selected without over-correcting hindfoot motion (Fig 3B). The MTS loading procedures were repeated identically with displacement, torque, and pressure measurements taken as described above after insertion of the appropriate sinus tarsi stent.

### Statistical analysis

**Fig 4:**
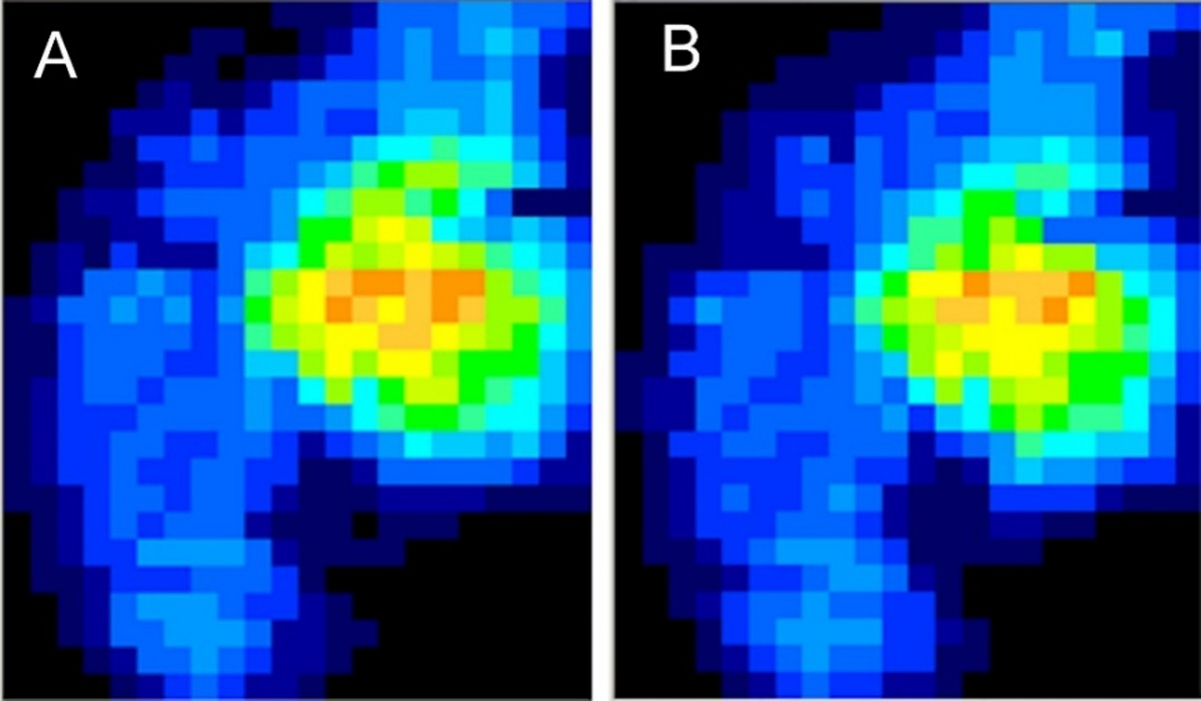
Force mapping of medial knee compartment forces.

The forces on the medial tibiofemoral joint were analyzed using the I-Scan software before and after the insertion of the sinus tarsi stent (Fig 4). The average force on each specimen over the entire loading period and the mean (SD) for all of the specimens were computed. Statistical analysis of the biomechanical data was performed by the two-tailed paired t-test using Sigstat software. Values are expressed as mean ± SD. A *p*-value < 0.05 was considered significant.

## Results

Our research shows that the transmission of force pre-EOTTS placement, onto the medial knee, as a result of the loss of alignment and stability of the talus on the tarsal mechanism, was an average of 842 ± 247N. The loading pattern for the medial compartment forces of the knee joint showed decreased joint forces for all ten specimens after the insertion of the sinus tarsi stent, with a mean force of 565 ± 260N (<0.05), or an average 32.8% overall reduction in medial compartment forces. The forces acting on the medial knee joint before and after the insertion of the sinus tarsi stent for each specimen are shown in Table 1.

**Table 1:** Comparison of medial compartmental compressive forces. Where Y = years, R = right limb, L = left limb, EOTTS = extra-osseous talotarsal joint stabilization, N = Newtons, SD = standard deviation.

Specimen	Age (Y)	Leg Side	Pre-EOTTS (N)	Post-EOTTS (N)	Stent Size
1	53	R	724	106	9
2	83	R	421	278	9
3	63	R	714	680	9
4	62	R	1219	716	8
5	60	R	1187	1113	8
6	49	L	708	525	8
7	67	R	792	621	8
8	95	R	672	380	9
9	68	R	1148	601	9
10	68	L	830	634	9
Mean (SD)	**66.8 (13.5)**		**841.5 (247.4) N**	**565.4 (273.7) N**	

## Discussion

This is the first study to evaluate the in vivo forces acting on the medial compartment of the tibiofemoral joint in limbs diagnosed with hindfoot instability, pre- and post-EOTTS. We theorized that EOTTS would decrease the forces acting on the medial knee compartment. Our hypothesis was confirmed with the results of our testing. EOTTS reduced medial knee forces by 32.8%. Decreasing these forces is clinically relevant when critically evaluating the efficacy of an intervention. This study helps to better explain a possible source of varus thrust and increased knee adduction moment. No prior study has evaluated the role or importance of EOTTS in decreasing the forces to the medial knee compartment.

The subtalar joint is the “foundation joint” of the body. Therefore, its alignment and stability can have a positive or negative effect on the superior structures. Only a short period of subtalar joint pronation, approximately 1/3^rd^ at the beginning of full plantar foot contact phase of the gait cycle, should exist [14]. The partial dislocation of the talus on the calcaneus leads to an unnatural unlocking of the hindfoot bones. This creates a prolonged instability of the foot referred to as hyperpronation, commonly called over-pronation. There has been a long association with foot hyperpronation and chronic knee pain [15,16].

The negative effects of a dynamic malalignment of the knee in the frontal plane leading to increased medial knee compartment forces and motion has been well demonstrated [17–19]. Ahlback found a 10 times propensity for medial compartment OA over lateral compartment [20]. The excessive medial compartment forces are created as the limb accepts weight in the stance phase of gait. Knee alignment will be less varus, more neutral, during lift-off or non-weightbearing, swing phase of gait [21].

Age is the major independent risk factor for the formation of OA [22]. Aging and OA are inter-related, not inter-dependent. The connection between age and OA has been unclear. A possible connection can be made by understanding the compounding micro-trauma that occurs within the body from the most common form of human locomotion, walking. The average person takes an estimated 80 million steps by 45 years of age. This could be a reason why the risk of developing OA substantially increases with each decade after the age of 45 years [23]. Osteoarthritic changes could evolve with even minor deviations from normal force or motion after tens of million steps are taken.

Andriacchi et al, showed that hindfoot instability shifts the loading patterns of the knee joint during the weightbearing portion of the gait cycle[24]. This phenomenon increases injury to cartilage, leading to degeneration. Resende et al, also showed a connection between knee osteoarthritis and a hyperpronating foot [25]. The posterolateral plantar aspect of the heel encounters the weightbearing surface. As the rest of the plantar foot makes contact, the talus internally rotates. Excessive pronation and plantar flexion of the talus leads to subtalar joint instability. This will cause the medial plafond of the tibia to dip inferiorly in the frontal plane. The medial femoral condyle will also drop and internally rotate as a result. Subtalar joint instability could also explain why supporting knee tissues, such as the ACL, are at an increased risk of damage. Studies have shown that ACL insufficiency is typically a precursor to knee OA [26]. Furthermore, it’s been discovered that ACL injury repair does not have a positive effect to delay knee OA [27].

EOTTS is a minimally invasive, internal gait modification treatment aimed at improving subtalar joint alignment [28,29]. It offers a conservative orthopedic option that has been shown to realign and stabilize the TTJ while still allowing a normal range of motion [30,31]. One of the many advantages of EOTTS is that the stent can be removed without any negative affect to the subtalar joint, unlike other osseous surgical procedures that are irreversible. Another advantage over lateral wedge insoles (LWI) is the fact that EOTTS is an internal option that does not have limitations related to patient compliance. The EOTTS device functions regardless if the patient is walking barefoot or in shoes. The importance of this internal option is highly advantageous over external measures, simply due to the fact of the limited ability of external measures to realign and stabilize the talus. Many authors have suggested that options other than direct knee surgery should be considered primary. This is due to the associated limitations, costs and risks with knee surgery.

There were a few limitations to this study. First, only the medial knee compartment forces were measured. The changes to the lateral compartment were not captured in this study, primarily due to the medial knee being the most commonly affected and to avoid introducing instability to the knee through dissection of the lateral capsule. We hypothesized that decreased forces on the medial compartment would result in the rebalancing of those forces and increasing the load borne by the lateral compartment. The mean force acting on the medial knee was 565 N and we theorize the remaining 435 N transferred to the lateral compartment, or through the patellofemoral joint. Further research could verify this hypothesis.

## Conclusion

The current study provides valuable data to a possible pathomechanical factor as a contributor to the development of knee OA. Our *in vivo* joint force measurements confirm the importance of realigning and stabilizing the subtalar joint. This is the first study to show that EOTTS, an internal gait modification treatment, provides a positive effect to decreasing the forces acting on the medial knee compartment. Rebalancing knee forces has been considered a primary goal of treatment. EOTTS should be considered a possible treatment option to prevent and treat chronic knee OA and pain. This data further helps to explain a possible reason why other forms of treatment have had limited long-term success. Further research to validate the patient reported outcomes and weightbearing radiographic analysis of EOTTS on patients with mild to severe knee OA are needed.
